# Combination Antibiotic Therapy for Orthopedic Infections

**DOI:** 10.3390/antibiotics14080761

**Published:** 2025-07-29

**Authors:** Eric Bonnet, Julie Lourtet-Hascoët

**Affiliations:** 1 Centre Régional d’Antibiothérapie, Service des Maladies Infectieuses et Tropicales, CHU de Toulouse, 31300 Toulouse, France; 2 Service de Microbiologie-Infectiologie, Hôpital Joseph Ducuing, 31300 Toulouse, France; 3 Service de Bactériologie, Hôpital Saint Antoine, 75012 Paris, France

**Keywords:** orthopedic infection, antibiotic combination, prosthetic joint infection, fracture-related infection, spinal implant infection, anterior cruciate ligament reparation infection, biofilm

## Abstract

**Background/Objectives**: Limited robust data support the use of antibiotic combinations in the treatment of orthopedic infections. However, in certain situations, the combination of antibiotics seems to be beneficial. This review aims to outline the circumstances under which a combination of antibiotics may be utilized in the treatment of orthopedic infections. **Methods**: We reviewed the existing guidelines on orthopedic infections and focused on situations where antibiotic combinations are recommended or proposed optionally. We chose vitro and animal studies that provide evidence for the effectiveness of several widely recommended combinations. **Results**: The combinations serve multiple purposes: they provide empirical coverage while awaiting microbiological results, offer targeted treatment for difficult-to-treat infections, and facilitate oral treatment primarily for staphylococcal infections. The objectives include enhancing bacterial coverage against Gram-positive and Gram-negative bacteria, achieving synergistic effects with bactericidal agents, and reducing the risk of antibiotic resistance. The review outlines specific combinations for fracture-related infections, periprosthetic joint infections, spinal infections, and anterior cruciate ligament reconstruction infections, emphasizing the importance of tailoring antibiotic choices based on local epidemiology and patient history. The review also addresses potential drawbacks of combination therapy, such as toxicity, higher costs, and drug interactions, underscoring the complexity of managing orthopedic infections effectively. **Conclusions**: According to the guidelines, several different proposals are made, depending in part on the countries’ epidemiology. In a well-defined situation, various authors propose either monotherapy or a combination of antibiotics. When a combination is suggested, the choice of antibiotics is based on the expected effect: broadening the spectrum, enhancing bactericidal activity, achieving a synergistic effect, or reinforcing biofilm activity to optimize the treatment.

## 1. Introduction

Antibiotic combinations refer to the concurrent use of two or more antibiotics to enhance therapeutic efficacy, broaden the antimicrobial spectrum, prevent resistance, or achieve synergistic effects in the treatment of infections. Such combinations are particularly relevant in managing complex infections, including biofilm involvement, such as osteoarticular infections. The effectiveness of these associations in improving the outcome of patients with osteoarticular infections has been demonstrated. Few studies in the literature consolidate findings on antibiotic combinations in orthopedic infections. Our objective is to provide a narrative (non-systematic) review outlining the indications for such combinations in these infections. Various national and international guidelines recommend the use of antibiotic combinations for treating different orthopedic infections.

These combinations are used first as empirical therapy while awaiting bacterial identification and antibiotic susceptibility results. Second, they serve as a first-line targeted treatment for certain difficult-to-treat bacteria. Third, they are employed for oral treatment of infections predominantly caused by staphylococci and occasionally mycobacteria (which will not be discussed in this review).

The objectives of antibiotic combination therapy can be multiple:

- To enhance bacterial coverage by employing broad-spectrum antibiotics that are effective against Gram-positive (aerobic) bacteria (including methicillin-resistant staphylococci), Gram-negative (aerobic) bacteria, and, sometimes, anaerobic bacteria. Broad-spectrum treatment is generally necessary for empirical treatment or in cases of documented polymicrobial infection.
- To add a bactericidal antibiotic such as an aminoglycoside, which is typically administered for at least 24 to 48 h in the case of severe sepsis or septic shock, to achieve a synergistic effect, particularly against staphylococci, enterococci, and certain Gram-negative bacilli infections.
- To enhance antibiofilm activity when utilizing rifampicin, for instance.
- To diminish the risk of resistance emergence to vulnerable antibiotics, including rifampicin, fusidic acid, fosfomycin, and even fluoroquinolones, when employed as anti-staphylococcal agents or fosfomycin and colistin when used to treat multi-resistant Gram-negative bacteria.

We will review these situations alongside the corresponding recommendations in the current guidelines. Conversely, some arguments could be made against the use of combinations: increased risk of toxicity, drug–drug interactions, ecological impact (selection of strains with multiple resistance mechanisms), higher costs, the need for venous access, and the risk of subsequent complications at or around the site of the implanted catheter.

## 2. Methodology

Experts in bone and joint infections, including infectious disease specialists, surgeons and microbiology specialists, examined the data concerning bone and joint infections. Infections after prosthesis implantation, fractures, spine instrumentation, and cruciate ligament reparation were retrieved by searching PubMed and provided a comprehensive review of current evidence. Articles in English published after 1980 were considered. Keywords included prosthetic joint infections, orthopedic, fractures, antibiotic combinations, spinal infections, implant, cruciate ligament infections, biofilm, and empirical treatment.

## 3. To Broaden the Spectrum of Antibiotic Treatment

Most orthopedic infections are monomicrobial, mainly due to staphylococci; however, in several series up to 25% of orthopedic infections are polymicrobial [1,2].

Only different Gram-positive species are often involved in polymicrobial infections, but an association of Gram-positive and Gram-negative bacteria is not rare. This implies that probabilistic treatment should usually cover staphylococci (including methicillin-resistant strains), streptococci, enterococci and Gram-positive anaerobes such as *Cutibacterium* spp. but also Enterobacterales and *Pseudomonas aeruginosa*.

Usual combinations include an antibiotic with in vitro activity against most Gram-positive bacteria (including methicillin-resistant staphylococci), which could be a glycopeptide (vancomycin rather than teicoplanin), a lipopeptide (daptomycin) or an oxazolidinone (linezolid or tedizolid), and a broad-spectrum beta-lactam (piperacillin-tazobactam, a third- or fourth-generation cephalosporin, or a carbapenem). All these combinations are also active against Gram-negative anaerobes (except those comprising a cephalosporin) and Gram-positive anaerobes, particularly *Cutibacterium* spp. The role of dalbavancin (or similar long-acting antibiotics) in treating orthopedic infections due to Gram-positive bacteria needs further clarification [3,4].

## 4. Probabilistic Treatment

### 4.1. Fracture-Related Infections (FRI)

The probabilistic treatment proposed for FRI could include amoxicillin-clavulanic acid or ampicillin sulbactam AND vancomycin (or another glyco/lipopeptide or an oxazodinone) [5].

The objectives are to propose a treatment active against MSSA, (beta-hemolytic) streptococci and anaerobes when using a combination of ampicillin or amoxicillin and a beta-lactamase inhibitor but also an agent active against methicillin-resistant staphylococci when using vancomycin (or similar anti-Gram-positive agents), particularly in case of carriage of MRSA or multiple surgeries or suspected low-grade infections. Of note, a beta-lactam/beta-lactamase inhibitor combination is not active against *Bacillus cereus* that are involved in early infections after open fractures, especially in rural areas and after war trauma, and cause consequent implant-associated infections [6].

However, vancomycin (or other anti-MRSA agents), which could be added, usually displays in vitro activity against *Bacillus cereus*.

High resistance rates to cephalosporins and cloxacillin/flucloxacillin among staphylococci have been reported [7,8]. In Eisner et al.’s study of Staphylococcus epidermidis isolates, 79.8% were resistant to beta-lactam antibiotic agents, and only 44% of the infecting organisms were susceptible to cefuroxime. In Sheehy et al.’s study, only 29% of isolates would have been treated with flucloxacillin.

Another notable bacterial species not included in the spectrum of the proposed probabilistic treatment is *Enterobacter cloacae*, which is present in the animal intestinal flora and, consequently, in soils, plants, and water. For patients with open fractures showing evident contamination by soil and plant fragments, the pressing question is whether these bacteria should be considered when selecting probabilistic antibiotic treatment. While microbiology differs across geographic areas (urban vs. non-urban), a recent study from a prominent British trauma center concluded that the empirical systemic antibiotic combination of teicoplanin and meropenem would have covered 96.3% of infection episodes [9,10,11]. However, these recommendations do not provide specific details on the agents to be selected against Gram-negative bacilli.

### 4.2. Periprosthetic Joint Infection (PJI)

The probabilistic treatments proposed for PJI include Vancomycin and a broad-spectrum beta-lactam (piperacillin-tazobactam, a third- or fourth-generation cephalosporin, or a carbapenem). The choice of the anti-Gram-negative agent could depend on the delay between prosthesis implantation and infection, the local epidemiology, and the likely hematogenous character of the infection [12,13].

For early post-operative infections, it is recommended to use a combination of glycopeptide or lipopeptide with cefepime, piperacillin-tazobactam, or carbapenem (meropenem or imipenem) [14,15,16].

This regimen effectively covers methicillin-resistant staphylococci, Enterobacterales, and *Pseudomonas aeruginosa*. Table 1 summarizes recommendations from expert groups and learned societies.

For late acute infections, Australian and New Zealand experts recommend cefazolin monotherapy if there are no septic signs or a combination of vancomycin and an anti-GNB agent if septic signs are present [17].

### 4.3. Spinal Infections

Probabilistic treatment proposed for spine implant-associated infection includes a broad-spectrum beta-lactam active against Gram-negative bacteria, such as piperacillin-tazobactam, associated with an antibiotic active against Gram-positive bacteria, including systematically methicillin-resistant staphylococci, such as vancomycin, for any post-operative spine infection or only for patients who had multiple previous surgeries [18,19].

The latter recommends ampicillin-sulbactam, in cases of first revision for the coverage of Gram-negative bacteria. Other studies showed that Gram-negative bacteria are increasingly involved in instrumented spinal infections, related to many elderly patients, and recommend possible alternatives such as piperacillin-tazobactam or cefepime associated with vancomycin, daptomycin, teicoplanin, Linezolid or Fosfomycin [20].

### 4.4. Anterior Cruciate Ligament Reparation Infection (ACLRI)

Probabilistic treatments proposed for ACLRI include ampicillin-sulbactam (or amoxicillin-clavulanic acid) and vancomycin or daptomycin [21]. In case of allergy (non-type 1) to penicillin, cefazolin or cefuroxime is suggested instead of penicillin-beta-lactamase inhibitor. In case of type 1 allergy to beta-lactams, daptomycin alone is suggested. These recommendations are supported by microbiological data showing that GNBs are rarely involved (<10%), whereas Gram-positive bacteria (including anaerobes such as *Cutibacterium* spp.) are present in over 90% of cases [21,22].

Antibiotics recommended in guidelines for probabilistic therapy are summarized in Table 1. (Combinations include an anti-Gram-positive and an anti-Gram-negative drug listed in Table 1.)

**Table 1:** Antibiotics suggested for empiric treatment of orthopedic infections.

	Type of Orthopedic Infection	FRI	PJIs	Spine Implant-Associated Infections	ACLR-Related Infections
Antibiotics					
Antibiotics active on Gram-positive bacteria					
Vancomycin		+ [11,15]	+ [15,16,17,23]	+ [18,19]	+ [21]
Teicoplanin		+ [23]	+ [23]	+ [18]	
Daptomycin		+ [23]	+ [16,23]	+ [18,19]	+ [21]
Linezolid			+ [23]	+ [18]	
Cloxacillin			+ [16]		
Amoxicillin-clavulanic acid ^a^			+ [23]		
Ceftriaxone ^b^			+ [23]		
3rd-generation cephalosporin + Fosfomycin ^c^		+ [15]	+ [15]		
Antibiotics active on Gram-negative bacteria					
Ampicillin-sulbactam or Amoxicillin-clavulanic acid ^a^				+ [19]	+ [21]
Cefazoline or Cefuroxime ^a^				+ [19]	+ [21] if non-type 1 penicillin allergy
Piperacillin-tazobactam		+ [10,15]	+ [15]	+ [18,19] only in case of multiple previous revisions	
Ceftriaxone or Cefotaxime		+ [15]	+ [15]		
Ceftazidime		+ [10]	+ [16]		
Cefepime		+ [10]	+ [16]	+ [15]	
Imipenem		+ [15]	+ [15]		
Meropenem		+ [15]	+ [15,16]		
Fosfomycin + Ceftriaxone ^c^		+ [15]	+ [15]		
Fosfomycin ^d^				+ [19]	

+ Recommended in national or international experts’ guidelines. Reference numbers are mentioned in parentheses. ^a^ Active against both Gram-positive bacteria (methicillin-susceptible staphylococci, streptococci, and enterococci (except for cefazolin and cefuroxime)) and, to a lesser extent, Gram-negative bacteria, and sometimes proposed in combination with vancomycin or daptomycin [19]. ^b^ non-active against methicillin-resistant staphylococci and enterococci. ^c^ Both antibiotics target Gram-negative and Gram-positive bacteria and can produce a synergistic effect against *E. faecalis*, *S. aureus* and some GNB [24,25,26,27]. ^d^ Proposed in combination with daptomycin, in case of multiple revisions. Vancomycin + piperacillin-tazobactam is an alternative.

### 4.5. Polymicrobial Infections

For pluri-microbial infections, the selection of antibiotics depends on susceptibility testing. Broad-spectrum antibiotics such as third-generation cephalosporins, piperacillin-tazobactam, or carbapenems can be used alone for Gram-negative bacilli and most Gram-positive cocci, including streptococci, methicillin-susceptible staphylococci, and *E. faecalis* (excluding cephalosporins). Piperacillin-tazobactam and carbapenems also cover Gram-negative anaerobes, negating the need for specific anti-anaerobic agents. Ceftaroline and ceftobiprole are alternatives for methicillin-resistant staphylococci [28,29].

## 5. To Enhance the Bactericidal Activity of the Initial Treatment

The term sepsis may sometimes be misused by doctors instead of infection. Sepsis is life-threatening organ dysfunction from a dysregulated response to infection, while septic shock involves severe abnormalities that increase mortality risk. Patients with orthopedic infections showing signs of sepsis or septic shock should receive broad-spectrum bactericidal antibiotics quickly.

Without prior bacteriological documentation or microbiological guidance, an aminoglycoside is often used for 24 to 48 h due to its rapid bactericidal activity against Gram-positive and negative bacteria. Aminoglycosides, particularly amikacin, can provide additional coverage against multi-resistant Gram-negative bacilli when used in combination with a glycopeptide or lipopeptide and a broad-spectrum beta-lactam.

## 6. To Achieve a Synergistic Effect for Targeted Treatment (See Table 2)

A synergistic effect may be necessary, especially at the start of treatment, for infections with staphylococci, enterococci, or Gram-negative bacilli such as *P. aeruginosa*. Although this synergistic effect has been primarily studied for treating bacteremia and in animal models, it is occasionally suggested for severe orthopedic infections despite a lack of convincing clinical data supporting this. Consequently, national and international guidelines include various recommendations.

**Table 2:** Proposed targeted antibiotic combinations in guidelines [10,15,16,18,19,21,23,30].

		IV ^a^	Oral ^b^
Staphylococci			
	MS	[(Cl or Dicl or Flux) Oxacillin or Cefazolin] + [Rifampicin ^c,d^ or Gentamicin ^e^ or Fosfomycin] If contraindications to beta-lactams: [Daptomycin or Vancomycin or Teicoplanin] + [fosfomycin or rifampicin ^c,d^ or gentamicin ^e^ or fusidic acid ^f^]	Levofloxacin (or ciprofloxacin or moxifloxacin) + Rifampicin Oral alternatives without quinolones: Rifampicin + [cotrimoxazole or clindamycin ^g^, minocycline (or doxycycline) or dicloxacillin or cefalexin or linezolid (or tedizolid) or fusidic acid] Oral alternatives without rifampicin: Levofloxacin + [linezolid (or tedizolid) or cotrimoxazole or fusidic acid] Linezolid + [cotrimoxazole or fusidic acid] ^h^ Clindamycin + fusidic acid
	MR	See IV combinations, listed above in the case of contraindications to beta-lactams.	Levofloxacin ^i^ (or Moxifloxacin) + Rifampicin Oral treatment without quinolones: see oral combinations listed above, except for dicloxacillin and cephalexin. Oral treatment without rifampicin: see oral combinations listed above.
Enterococci			
	Penicillin G (or amoxicillin)-S	[Penicillin G or Ampicillin or Amoxicillin] + [Ceftriaxone or Gentamicin (+ Fosfomycin) or Rifampicin ^c,d^]. If contraindication to beta-lactam: [Vancomycin or Teicoplanin or Daptomycin or Linezolid] + [Gentamicin (+ Fosfomycin) or Rifampicin ^c,d^]	Amoxicillin + rifampicin
	Penicillin G (or amoxicillin)-R	See IV combinations, listed above in the case of contraindications to beta-lactams.	Linezolid + rifampicin
Streptococci		Amoxicillin (or Ceftriaxone) + Gentamicin ^j^ If contraindication to beta-lactam: [Vancomycin or Teicoplanin or Levofloxacin] + [Gentamicin or Rifampicin] ^c,d,j^	Amoxicillin + Rifampicin ^j^ If contraindication to beta-lactam: [Levofloxacin or Clindamycin ^g^] + Rifampicin ^j^
		IV ^a^	Oral ^b^
Enterobacterales		Beta-lactam ^j,k^ [Ceftriaxone or Cefotaxime or Piperacillin-tazobactam or Cefepime ^l^ or Imipenem ^m^ or Meropenem ^m^] + [Aminoglycoside (Gentamicin or Tobramycin or Amikacin) or Fluoroquinolone (Ciprofloxacin or Levofloxacin) or Fosfomycin ^n^ and/or Colistin ^n^]	*Monotherapy*
*P. aeruginosa*		[Cefepime or Ceftazidime or Piperacillin-tazobactam or Meropenem or Imipenem] + [Aminoglycoside (Gentamicin or Tobramycin or Amikacin) or Fluoroquinolone (Ciprofloxacin or Levofloxacin) or Fosfomycin ^n^ and/or Colistin ^n^]	Monotherapy
*Cutibacterium acnes* and other Gram+ anaerobes		Monotherapy	[Levofloxacin or Doxycycline] + rifampicin ^j^
Gram− anaerobes		Monotherapy	Monotherapy

^a^ As initial treatment, or when the oral route for maintenance therapy is not feasible, or, sometimes, in the case of an allergy to beta-lactams. ^b^ Typically administered as maintenance therapy following an initial intravenous treatment lasting one to six weeks (according to the guidelines), typically one to two weeks. ^c^ Most authors suggest delaying the introduction of rifampicin because of the high risk of selection of resistant mutants due to the high inoculum. ^d^ If possible, rifampicin should be administrated orally because of its high bioavailability; however, authors advocate administering it intravenously due to poor digestive tolerance of pills. ^e^ As suggested for staphylococcal endocarditis, recent guidelines for orthopedic infections do not recommend using combinations with gentamicin (or other aminoglycosides) due to their nephrotoxicity. ^f^ Preferably by oral route. ^g^ Rifampicin lowers the plasma concentration of clindamycin; therefore, it is advisable to monitor clindamycin levels (see in text). ^h^ Theoretical enhancement of hematotoxicity risk. ^i^ Most methicillin-resistant *S. aureus* are also resistant to quinolones; however, the link between methicillin and quinolone resistances is significantly weaker for coagulase-negative staphylococci. ^j^ Most of guidelines suggest monotherapy with a beta-lactam (amoxicillin for *C. acnes*). Alternatives: levofloxacin or ciprofloxacin for GNB and clindamycin for *C. acnes*. ^k^ Some guidelines do not provide details on which beta-lactam to choose. ^l^ For *Enterobacter cloacae* and other Enterobacterales at risk of amp-C production (derepressed cephalosporinase). ^m^ For ESBL Enterobacterales. ^n^ For multi-resistant GNB.

### 6.1. Staphylococcal Infections

Guidelines generally suggest using a single anti-staphylococcal beta-lactam for methicillin-susceptible strains. They recommend a glycopeptide or a lipopeptide and occasionally cotrimoxazole, linezolid, and minocycline for methicillin-resistant strains [16,18,19,21,23,30].

When combination therapy is suggested, often optionally, the choices for a second agent include gentamicin, fosfomycin, rifampicin, daptomycin (with cloxacillin for methicillin-susceptible strains), or cloxacillin (with daptomycin for methicillin-resistant strains) [16,18,19,23].

### 6.2. Enterococcal Infections

As the modal penicillin MIC is higher than that for streptococci, adding an aminoglycoside (usually gentamicin) to penicillin is common, as recommended for enterococcal endocarditis [31,32,33,34]. However, few solid data support this combination for enterococcal orthopedic infections. Another synergistic treatment for enterococcal endocarditis involves using ampicillin or amoxicillin in combination with a third-generation cephalosporin. This synergy has also been documented when using second- or fourth-generation cephalosporins [35].

Enterococcal orthopedic infections are challenging to treat, making synergistic antibiotic combinations appealing. Guidelines for diagnosing and treating prosthetic joint infections and implant-associated infections suggest adding gentamicin or ceftriaxone (and optionally fosfomycin) to penicillin if the *Enterococcus* sp. is penicillin-susceptible [15,16,18,19,21].

For infections caused by Enterococcus that are resistant to penicillin or for patients who are allergic to penicillin, it is recommended to use a combination of vancomycin or teicoplanin or high doses of daptomycin and gentamicin [15,16,18,19,21]. In some cases, additional Fosfomycin may also be considered.

Spanish guidelines for PJI management suggest adding ceftriaxone to ampicillin as optional, while IDSA guidelines recommend using just ampicillin [5,16,30].

### 6.3. GNB

Antibiotic combination therapy is generally not recommended for Enterobacterales, except according to the French guidelines published in 2009. These guidelines suggest combining a broad-spectrum beta-lactam (such as cefotaxime or ceftriaxone, or a carbapenem if the others are ineffective) with either a quinolone (such as ofloxacin or ciprofloxacin) or gentamicin. However, an update on this recommendation is currently being processed [15].

The topic of antibiotic combinations is particularly relevant for non-fermenters (*Pseudomonas aeruginosa* and *Acinetobacter species*). In Spanish, Italian and IDSA guidelines, monotherapy is proposed for all Gram-negative bacilli, including *P. aeruginosa* [16,23,30,36].

The pro-implant foundation’s proposals for treating non-fermenting Gram-negative bacilli, as discussed in the paper by Pawloski et al., suggest using a broad-spectrum beta-lactam (such as piperacillin-tazobactam, meropenem, or ceftazidime) in combination with an aminoglycoside (like tobramycin or gentamicin) [37].

However, due to the risk of nephrotoxicity associated with aminoglycosides, and considering the penetration of fluoroquinolones into bone and joint tissues as well as their effectiveness against non-fermenting bacilli, ciprofloxacin may be regarded as a suitable alternative.

For multi-drug-resistant Gram-negative bacilli, newly developed beta-lactamase/beta-lactam combinations and cefiderocol might represent the only viable treatment options. Nevertheless, additional research is needed to evaluate their efficacy in orthopedic infections [38].

Old antibiotics such as colistin or Fosfomycin, in combination with beta-lactams or other agents (including new beta-lactamase/beta-lactam combinations), could act synergistically and represent valuable options [39,40,41,42].

## 7. To Reinforce Antibiofilm Activity (See Table 2)

As the antibiofilm activity of most antibiotics used to treat Gram-positive orthopedic infections is low (if present), rifampicin is often proposed as an additional agent [43,44,45,46,47].

For staphylococcal PJI, authors recommend starting rifampicin immediately after surgical treatment (or even perioperatively) in combination with (flu)cloxacillin or cefazolin in the case of a methicillin-susceptible strain, or vancomycin or daptomycin in the case of a methicillin-resistant strain. However, others suggest waiting a few days before introducing rifampicin, arguing that the low local concentration of the combined antibiotic provides insufficient protection against the selection of resistant strains. Rifampicin is not optimal during the first few days of treatment. The best companion for rifampicin appears to be fluoroquinolones, and this combination has been recommended in numerous guidelines or consensuses [15,18,19,21,36]. Other antibiotics advised for use alongside rifampicin include clindamycin, cotrimoxazole, tetracyclines, and linezolid, with insufficient data to compare them.

However, despite these findings [48,49,50,51], clinical studies have not observed poor outcomes when clindamycin was combined with rifampicin to treat orthopedic infections [50,52].

Recent studies suggest that to achieve adequate clindamycin concentrations, clindamycin should be used only at high doses via continuous IV administration when combined with rifampicin [48,53,54,55].

Despite conflicting clinical data regarding the effectiveness of the linezolid and rifampicin combination [56], its use is recommended in various guidelines and advocated by several authors, primarily for treating methicillin-resistant staphylococci infections [15,16,18,36]. Moreover, the hematologic toxicity of linezolid appears to be diminished when combined with rifampicin [57].

Other members of the rifamycin family, such as rifabutin and rifapentine, could serve as interesting alternatives to rifampicin, as they may exhibit similar or even greater in vitro activity than rifampicin. They also demonstrate antibiofilm and intracellular activity in staphylococcal infections while producing fewer adverse events and drug interactions [58,59,60,61].

There are compelling results from animal models and promising clinical data [62,63,64]. Clinical trials should be encouraged to evaluate rifabutin as a substitute for rifampicin in staphylococcal periprosthetic joint infections. The combination of rifampicin with other antibiotics for treating infections caused by different bacteria (streptococci, enterococci, *Cutibacterium* sp.) has yet to be established. Furthermore, recent studies have reported no clinical benefit in adding rifampicin to other antibiotics for treating orthopedic infections caused by *Cutibacterium* sp. [65,66,67,68].

The value of combining rifampicin with beta-lactams or other antibiotics for treating streptococcal or enterococcal orthopedic infections remains unclear [69,70,71,72]. The use of rifampicin in mycobacterial infections will not be covered here. Many questions remain concerning the practical use of rifampicin: Is it necessary after any surgical strategies? When should it be commenced? What is the optimal daily dose and mode of administration? How long should it be maintained? These points will not be discussed here.

Fluoroquinolones demonstrate superior activity against biofilms compared to other antibiotics and are more effective in both animal models and humans against device-associated Gram-negative bacilli (GNB) infections [45,73,74,75]. A synergistic effect of fosfomycin, ciprofloxacin, and gentamicin in combination has been demonstrated in animal models against *Escherichia coli* and *Pseudomonas aeruginosa* biofilms [76]. However, no clinical data supports the results reported in experimental study models. Therefore, quinolones should be utilized as monotherapy whenever feasible for GNB-related orthopedic infections.

## 8. To Reduce the Risk of Resistance Emergence (See Table 2)

Some antibiotics, when used alone, are especially susceptible to the emergence of resistant strains. The risk also depends on the type of bacteria.

### 8.1. Gram-Positive Cocci

The risk of selecting drug-resistant bacteria is well documented when quinolones are used alone for treating staphylococcal infections. The most studied partner is rifampicin, owing to its advantages mentioned above for treating staphylococcal orthopedic infections [43,77,78,79].

There is less information regarding the combination of levofloxacin with other antibiotics besides rifampicin [80].

Other antimicrobial options for managing orthopedic staphylococcal infections include rifampicin (see above), fosfomycin, and fusidic acid [81,82,83,84,85]. However, when used alone, these agents present a significant risk of rapidly developing resistance. Thus, it is crucial to combine them with another antibiotic when treating severe staphylococcal infections. In cases of BJI caused by MRSA and enterococci, the combination of daptomycin and ceftobiprole may help reduce the likelihood of developing resistance and infection relapse [86,87].

### 8.2. Gram-Negative Bacilli

For non-resistant Enterobacterales (i.e., susceptible to third-generation cephalosporins), there is no need for antibiotic combinations; monotherapy with a third-generation cephalosporin or, if active, a fluoroquinolone (ciprofloxacin or levofloxacin) appears to be sufficient. However, during the first post-operative days (5–7 days), European guidelines recommend a combination of a beta-lactam and an aminoglycoside, fluoroquinolone, or, even optionally, fosfomycin or colistin [10,15,16,18,37].

Carbapenems, whether used alone or in combination (like other mentioned beta-lactams), should be reserved for infections caused by BLSE, non-CRE Enterobacterales. For CRE, the recommended treatment options include cefiderocol or a fixed regimen of a beta-lactam combined with a beta-lactamase inhibitor. The resistance mechanism determines the choice of the combination: aztreonam-avibactam is effective against all types of CRE, while ceftazidime-avibactam targets Oxa48 and KPC-producing Enterobacterales but not strains with metallo-beta-lactamases. Additionally, imipenem-relebactam and meropenem-vaborbactam are effective only against KPC-producing strains. There is currently limited data on the use of new beta-lactam/beta-lactamase inhibitor combinations for treating osteoarticular infections caused by GNB. In any case, for patients with CRE infections that are susceptible and treated with a beta-lactam/beta-lactamase inhibitor or cefiderocol, adding another agent is not advised [36,88].

For non-fermenter GNB (*P. aeruginosa*, *Acinetobacter* spp.), most guidelines propose a combination of a beta-lactam (ceftazidime, cefepime, piperacillin-tazobactam, imipenem, or meropenem) and an aminoglycoside (amikacin, tobramycin, or even gentamicin), or ciprofloxacin, or Fosfomycin [15,16,21,37].

The duration of the combined therapy often reaches two weeks. Ciprofloxacin is the recommended fluoroquinolone for treating pseudomonal infections, while amikacin and tobramycin are the preferred aminoglycosides. It has not been established that a carbapenem is more effective than other beta-lactams for treating infections caused by *P. aeruginosa* strains susceptible to all antipseudomonal antibiotics. To date, excluding cases where combination therapy is recommended (such as treatment with colistin, aminoglycosides, or fosfomycin), there is insufficient evidence to support the use of dual therapy for treating *P. aeruginosa* infections, including severe cases [36,88].

Nonetheless, given the limited data on the effectiveness of combining various antibiotics with antipseudomonal beta-lactams, dual therapy warrants consideration in complex infections. Beta-lactams can be combined with fluoroquinolones, aminoglycosides, fosfomycin, and colistin. As previously mentioned, using aminoglycosides, fosfomycin, and colistin as monotherapy for treating Gram-negative bacterial bone and joint infections should be strongly discouraged due to the high risk of developing resistant strains.

Although animal studies demonstrate promising results, the effectiveness of combination therapy for orthopedic infections remains uncertain in many cases and necessitates further research.

Despite the use of antibiotic combinations in orthopedic infections, it faces substantial limitations. Increasing antimicrobial resistance, particularly among Gram-negative bacteria or coagulase-negative staphylococci, diminishes the effectiveness of standard regimens [36,88]. The ability of bacteria to form biofilms on implants shields them from both antibiotics and immune responses, further complicating the treatment of combinations [89]. Clinical evidence remains heterogeneous, making it difficult to define optimal protocols. Toxicity is a concern (combinations like vancomycin and aminoglycosides) that can elevate risks of nephrotoxicity, especially in older populations [90]. Additionally, culture-negative infections hinder the tailoring of therapy, particularly in cases of previous antibiotic exposure [91]. Moreover, the limited penetration of some antibiotics in bone and joint tissue shows suboptimal activity in infected tissues [92].

Recent developments in the field of antibiotic combinations for bone infections aim to overcome resistance, improve delivery, and enhance patient outcomes.

Building on the OVIVA trial, recent studies continue to support the use of oral antibiotics as effective alternatives to prolonged IV therapy in bone and joint infections (BJIs) [93]. This study has an impact on the reduction of hospital stays, costs, and catheter-related complications.

Recent antibiotic options like dalbavancin and oritavancin are efficient to maintain therapeutic levels with infrequent dosing [94]. The use of local antibiotic delivery systems, such as antibiotic-loaded cement beads or spacers, is an effective complement to systemic antibiotics. These devices deliver high concentrations of antibiotics directly to the site of infection, which can improve bacterial eradication and promote bone healing [95].

Novel strategies have emerged as a potential strategy for eliminating infections, particularly in refractory situations. Research into bacteriophage therapy has the potential to improve treatment outcomes [96,97], with naturally occurring viruses that specifically infect bacteria leading to lysis of the bacterial cell wall. It has emerged as a promising approach for treating FRI [98]. The use of vaccines also shows promise for prevention and therapeutic intervention [99,100].

## 9. Conclusions

Antibiotic combinations remain a critical component in the management of orthopedic infections, especially regarding antimicrobial resistance and biofilm-associated challenges. While traditional regimens have demonstrated utility, their limitations—ranging from toxicity and resistance development to inconsistent clinical outcomes—underscore the need for more targeted and innovative approaches. Recent advances, including long-acting agents, novel delivery systems, and adjunctive therapies like phage treatment, offer promising alternatives that may enhance efficacy and reduce systemic burden. However, the heterogeneity of infections and patient profiles demands individualized, multidisciplinary strategies. Future research should focus on optimizing combination protocols, integrating emerging therapies, and refining diagnostic tools to guide precision treatment.
